# Concentration changes in gemcitabine and its metabolites after hyperthermia in pancreatic cancer cells assessed using RP-HPLC

**DOI:** 10.1186/s11658-019-0153-1

**Published:** 2019-05-16

**Authors:** HB Jin, L Lu, L Xie, JF Yang, XF Zhang, SL Ma

**Affiliations:** Department of Gastroenterology, Affiliated Hangzhou First People’s Hospital, Zhejiang University School of Medicine, Hangzhou, 310006 China

**Keywords:** Pancreatic cancer, Gemcitabine, Metabolite, Hyperthermia, RP-HPLC

## Abstract

**Background:**

Gemcitabine (2′,2′-difluoro-2′-deoxycytidine;dFdC) is a first-line chemotherapy drug for pancreatic cancer. Recently, a synergistic anti-tumor treatment of dFdC and hyperthermia has achieved good clinical results, but there are few reports on the molecular mechanism influenced by hyperthermia. This study is an initial exploration of the effects of hyperthermia on changes in the concentration of dFdC and its metabolites in pancreatic cancer cells. The aim is to provide a theoretical basis for clinical detection and pharmacokinetic research.

**Methods:**

PANC-1 cells at logarithmic growth phase were used as the experimental object. The MTT assay was performed to determine the half maximal inhibitory concentration (IC_50_) of dFdC. After PANC-1 cells were cultured in DMEM medium containing IC_50_dFdC and treated with hyperthermia at 41 °C or 43 °C, changes in the concentration of dFdC, 2′,2′-difluorodeoxyuridine (dFdU) and difluorodeoxycytidine triphosphate (dFdCTP) in the cells were tested using an optimized reverse phase high-performance liquid chromatography (RP-HPLC) protocol.

**Results:**

We found that 41 °C and 43 °Chyperthermia gave rise to a decrease in dFdC and dFdU content. At 41 °C, the levels respectively fell to 9.28 and 30.93% of the baseline, and at 43 °C, to 24.76 and 57.80%, respectively. The dFdCTP content increased by 21.82% at 41 °C and 42.42% at 43 °C.

**Conclusion:**

The two heat treatments could alter the mechanism of dFdC metabolism in PANC-1 cells. The effect of 43 °C hyperthermia is more significant. Our observations may be instrumental to explaining the higher anti-tumor efficacy of this combination therapy.

## Background

Pancreatic cancer is one of the most common types of malignant tumor. It is a leading cause of cancer-associated mortality worldwide, with a 5-year overall survival rate of less than 5% [1, 2]. Recent studies have predicted that by 2030, its fatality rate will rise to second place in the United States (after non-small cell lung cancer) [3]. Its incidence is also increasing annually. Tumor resection is the only available curative treatment, but approximately 80% of patients are ineligible for surgery due to an unfavorable tumor location and/or metastases [4]. Chemotherapy and radiotherapy are applied to improve these poor results [5].

Gemcitabine (2′,2′-difluoro-2′-deoxycytidine; dFdC), a pyrimidine nucleoside analog, is a cell cycle-specific anti-cancer chemotherapy drug approved by the US Food and Drug Administration (FDA). It has a broad spectrum of antitumor activity [6]. It is also an active radio-sensitizer [7]. A randomized trial proved it has greater clinical and survival benefits than5-fluorouracil [8]. However, there are still some problems in its clinical applications, such as its low response rate, the rate degradation of the active substance, and tumor resistance [9, 10]. The chemotherapy using dFdC combined with albumin-bound paclitaxel has been approved internationally [11, 12] and could significantly prolong the median survival time, reduce side effects and improve the quality of life for patients [13]. However, the wide application of this regimen is mainly limited by the high price of albumin-bound paclitaxel.

Therapeutically applied hyperthermia uses physical heating to maintain the temperature of tumor tissues at 40–44°Cfor a period of time. It can lead to growth block or death of the tumor cells without damage to normal cells. Heat treatment has recently attracted more attention due to its remarkable biological efficacy in the treatment of tumors and the advantages of being painless, non-invasive and without side effects [14].

Several studies have consistently demonstrated that some chemotherapeutic agents possess a synergistic anti-tumor efficacy when combined with regional hyperthermia [15–18]. dFdC combined with hyperthermia has been widely used in clinical practice. The clinical results for dFdC used in hyperthermic intraperitoneal intraoperative chemotherapy (HIPEC) in patients with resectable pancreatic cancer appear very promising [19]. Hyperthermia can induce Hsp70 to inhibit the activation of nuclear factor kappa B (NF-*κ*B), thereby increasing the toxicity on pancreatic cancer cells [20]. Past research also demonstrated that hyperthermia increased the arrest of S phase and the apoptosis of pancreatic cancer cells by causing the over-expression of heat-shock protein 27(Hsp27) [21].

It has now been recognized that the metabolic pathway of dFdC is similar to that of cytarabine [22]. dFdC is transported into the intracellular region via human equilibrative nucleoside transporters 1 (hENT1) [23, 24], and catalytically converted to dFdCmono-, di- and triphosphate by deoxycytidine kinase. These forms subsequently block ribonucleotide reductase and DNA synthesis [25, 26]. In addition, dFdC can undergo an irreversible deamination reaction under the action of cytidinedeaminase and generate the inactive metabolite 2′,2′-difluorodeoxyuridine (dFdU), which is excreted outside the cell later. Alternatively, dFdCMP produces difluoromonophosphate deoxyuridine via 2′-deoxycytidine 5′-monophosphate (dCMP) deaminase, and then creates dFdU [27]. Therefore, some methods for detecting the concentration of dFdC metabolites and studying pharmacokinetics have been established [28], among which reverse phase high-performance liquid chromatography (RP-HPLC) was the most efficient and sensitive [29–31].

There are few reports on the effects of hyperthermia on the metabolic mechanism of dFdC in pancreatic cancer cells, and related detection methods are urgently needed for further optimization of the treatment. In this study, we used reverse phase high-performance liquid chromatography (RP-HPLC) to test the concentration changes of metabolites in pancreatic cancer cells (PANC-1) after treatment with dFdC combined with hyperthermia. Furthermore, we made an initial exploration of the effects of hyperthermia on the metabolism. Our aim was to provide a reference for the study of its pharmacokinetic mechanism and clinical drug detection.

## Methods

### Cell culture and hyperthermia pretreatment

Stored PANC-1 cells (purchased from the Institute of Biochemistry and Cell Biology, Shanghai Institutes for Biological Sciences, Chinese Academy of Sciences) were thawed quickly in 37–40 °C water. The cell fluid was centrifuged for 5 min at 1000 rpm to separate the supernatant, which was then discarded. The precipitate was added to the culture medium, which was Dulbecco’s modified Eagle medium (DMEM) containing 10% fetal bovine serum and 1% antibiotic, then mixed well by gentle blowing. Further centrifugation under the same conditions was used to separate the supernatant again. After the second precipitate was dissolved well in the culture medium, it was transferred into a 25-cm^2^ cell culture flask and cultured in a saturated humidity incubator at37 °C and 5% CO_2_.The cells were digested with 0.25% trypsin containing 0.02% EDTA (Hangzhou Lanbao Biotechnology Service) when they reached 80% coverage of the bottom of flasks. They were then subcultured into a 24-well plate at a concentration of ~ 2 × 10^5^ cells/ml in the above incubator. Those in thelogarithmic growth phase were selected as experimental subjects. To simulate the hyperthermia treatment, PANC-1 cells were incubated in a water bath at 41 °C or 43 °Cfor 1 h.

### Determination of the appropriate concentration of dFdC via MTT assay

Culture mediums based on DMEM were prepared under aseptic conditions with different concentrations of dFdC (2.5, 5.0, 10.0, 20.0 and 40.0 μg/ml). PANC-1 cells were inoculated into 96-well culture plates in an incubator at 37 °C and 5% CO_2_.After the monolayer culture had covered the whole bottom of wells, the original culture medium was replaced with the above DMEM-based one. Cells from the blank control group were still cultured in the original culture medium. Simultaneously, the treated groups of 41 °C and 43 °Chyperthermiawere also established.

After culture for 2 h, the culture medium was discarded. The remains were washed with phosphate-buffered saline (PBS) 2 or 3 times, then MTT solution (5 mg/ml, pH 7.4) was added to all wells followed by an additional 4 h incubation. Then, the mixture was added to dimethyl sulfoxide solution along and shaken for 10 min on a shaker at a low speed to fully dissolve the crystals. Absorbance was measured with a Molecular Devices plate reader at 490 nm. The cell growth inhibition ratio was calculated with the formula.

$$ \mathrm{Cell}\ \mathrm{growth}\ \mathrm{inhibition}\ \left(\%\right)=\left(1-\mathrm{drug}\ \mathrm{treatment}\ {\mathrm{A}}_{490}/\mathrm{control}\ \mathrm{group}\ {\mathrm{A}}_{490}\right)\times 100\% $$

### RP-HPLC detection of dFdC and its metabolites at different temperatures

#### Chromatographic conditions

RP-HPLC was performed with a Shimadzu 20AT HPLC System with a Zhida N2000 Chromatography Workstation at Zhejiang University. The main column was an Agilent TC-C_18_(2) (5 μm, 4.6 × 150 mm) and the guard column was an Agilent TC-C_18_(2) (5 μm, 4.6 × 12.5 mm). The mobile phase was acetonitrile (0.01 M ammonium acetate at pH 6.5 and 2:98 v/v); the flow rate was0.8 ml/min; the column temperature was30°C; the UV detection wavelength was268 nm; and the injection volume was20 μl.

#### Preparation of stock and working solutions

Stock solutions (1 mg/ml) of dFdC, dFdU and dFdCTP were prepared by dissolving standard drugs in methanol and ultra-pure water, respectively, and were stored at − 20 °C for further use. For the internal standard (IS), 3 mg/ml stock solutions of cefaclor were prepared by adding acetonitrile to the solute and mixing well. Working solutions (100 μg/ml) of all the standards were prepared each time by diluting corresponding stock solutions in afore mentioned solvents.

#### Isolation and purification of cell lysate

The harvested cell lysate was blended with IS solution (1.0 mg/ml). Then 0.1 ml chromatographic acetonitrile was added to the mixture. After it was centrifuged at 18,000 rpm for 5 min, the upper organic phase was isolated and mixed well with 0.1 ml n-hexane. After centrifuging again under the same settings, the components in the upper layer were removed. These isolation and purification procedures were repeated twice. The samples in the lower acetonitrile layer were further executed for RP-HPLC detection.

#### Establishment of calibration curves

Working solutions of dFdC, dFdUand dFdCTP were separately serially diluted in cell lysate from the control group to prepare 6 calibration solutions (20.0, 10.0, 5.0, 2.5, 1.25 and 0.5 μg/ml). After the isolation and purification pretreatment, all the target standards were examined in duplicate using the RP-HPLC assay. The average peak area was analyzed using the linear least square regression method. Calibration curves were generated simultaneously, with assessments of the correlation coefficient (r^2^*)* and slope.

#### Recovery and precision inspection

Three different concentrations (0.5, 5.0and 10.0 μg/ml) within the calibration range were prepared using standard working solutions to evaluate recovery and precision of this method. Each sample was tested and analyzed 5 times on the same day and on three consecutive days to determine intra- and inter-day precision, respectively. The percentage of recovered compound was also established from these quality control (QC) samples.

#### Calculation of LOD and LOQ

For three target compounds, limit of detection (LOD) and limit of quantification (LOQ) were determined by dividing the standard deviation (σ) of the Y-intercepts of different linear regression equations with slopes, applying the following relationship: LOD = 3.3 × σ/slope and LOQ = 10 × σ/slope.

#### Cell dosing and metabolite detection

PANC-1 cells in the logarithmic growth phase were cultured for 2 h with DMEM containing 14.073 μg/ml dFdC in an incubator at 37 °C and 5% CO_2_.Groups of 37 °Ccontrol and 41 °Cand 43 °C hyperthermia were established synchronously. The entire culture medium was discarded and the reproductive cells were washed once in the blank DMEM. The isolation and purification of samples was performed immediately after complete lysis of the cells using lysis reagent. Then, 20 μl purified solution was subjected to RP-HPLC analysis. After the chromatograms of three drugs were acquired, their peak area ratios were substituted into the corresponding equations for the calibration curves to calculate the contents of dFdC, dFdU and dFdCTP.

## Results and discussion

### Optimal concentration of dFdC for combination with hyperthermia

**Fig. 1:**
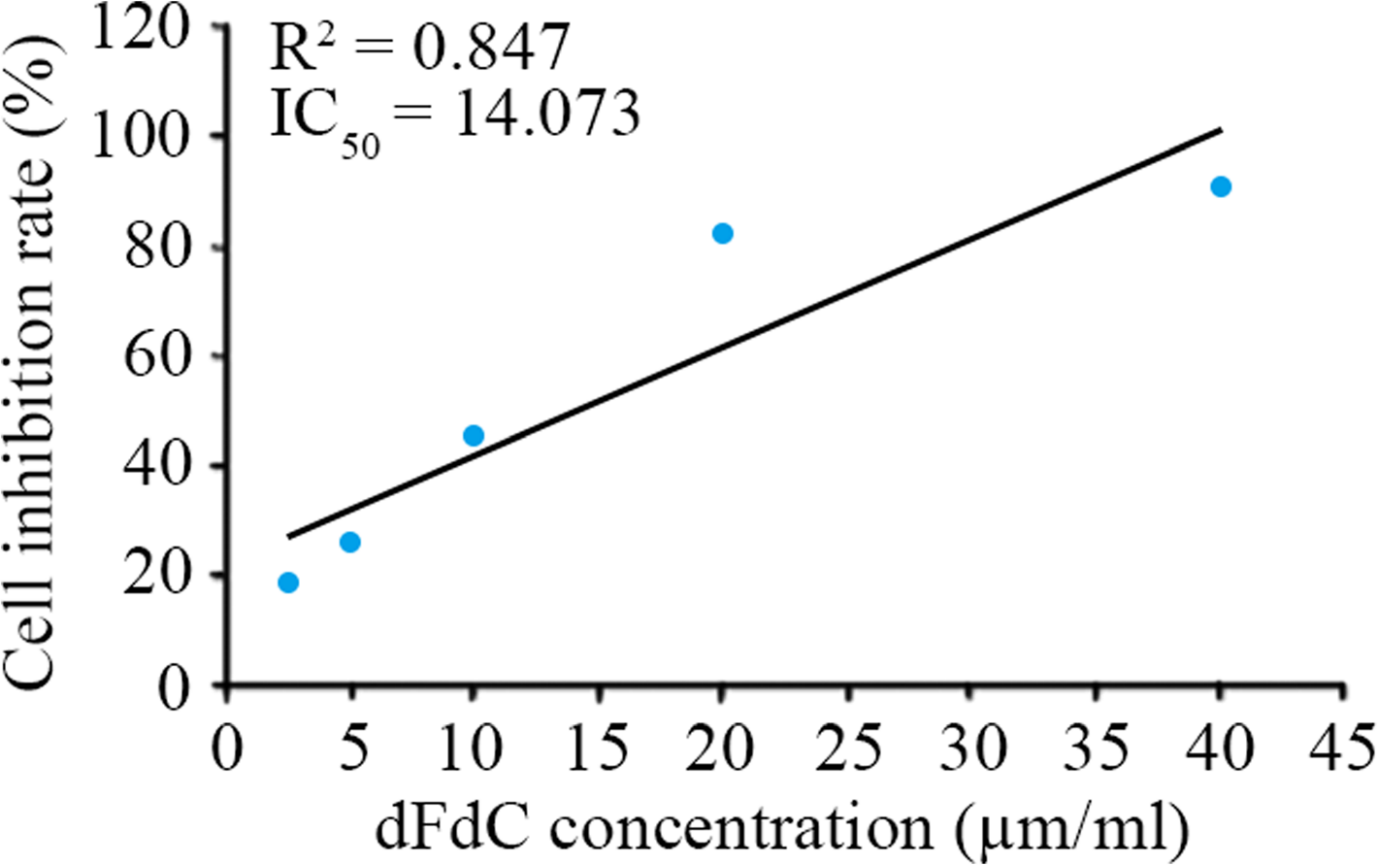
A scatter plot of the response of dFdC (gemcitabine) concentration (x) respond to the inhibition rate (y) as calculated with the regression equation y = 1.9829x + 22.095

After treating PANC-1 cells with different concentrations of dFdC combined with both hyperthermia conditions (41 °C and 43 °C), changes in the absorbance and cell inhibition ratios were assessed with the MTT assay (Table 1). Based on the linear regression analysis, the equation for the concentration (x) and inhibition rate (y) of dFdC was obtained (Fig. 1). When the cell inhibition was 50% (i.e., IC_50_), we calculated out the corresponding dFdC concentration (14.073 μg/ml). This concentration was applied to further study the metabolic process of dFdC combined with hyperthermia in PANC-1 cells.

**Table 1:** Absorbance and cell inhibition ratios of different concentrations of dFdC combined with hyperthermia as assessed with the MTT assay

Concentration (μg/ml)	Absorbance						Average value	SD	Inhibition ratio (%)
	37 °C		41 °C		43 °C				
0	0.692	0.637	0.854	0.802	0.768	0.708	0.744	0.079	
2.5	0.594	0.601	0.713	0.612	0.524	0.584	0.605	0.062	18.67
5.0	0.608	0.594	0.537	0.553	0.489	0.518	0.550	0.045	26.05
10.0	0.336	0.337	0.484	0.506	0.417	0.358	0.406	0.075	45.35
20.0	0.105	0.127	0.156	0.136	0.118	0.127	0.128	0.017	82.76
40.0	0.089	0.091	0.062	0.055	0.042	0.048	0.065	0.021	91.32

### Optimization of the sample pretreatment process

Many extraction reagents were assessed during the process of sample pretreatment. Methanol can precipitate proteins very well, but the supernatant obtained from the mixture showed trace proteins when stored at 4 °C overnight, which can lead to an increase in column pressure. Methylene chloride has the advantage of low pH and boiling point, but its solubility rate for dFdC and its metabolites was extremely low. The precipitation efficiency of trichloroacetic acid was the highest, but dFdC and its metabolites canbe decomposed heavily by this reagent. Acetonitrile enabled better precipitation of the target proteins, its solubility rate for dFdC and its metabolites was high, and it did not decompose them. Therefore, acetonitrile was recognized as the optimized choice.

### Characteristics of the internal standard and target chromatographic peaks

**Fig. 2:**
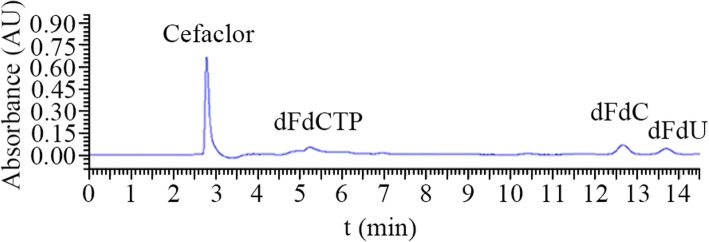
Representative chromatograms of cefaclor (the internal standard), and dFdC, dFdU and dFdCTP at 268 nm

Cefaclor was used as internal standard because of its stable and moderate retention time (~ 2.744 min) and good separation effect (Fig. 2). The chromatographic peak analysis shows that the retention times of dFdC, dFdU and dFdCTP were about 12.401, 13.565 and 5.213 min, respectively (Fig. 2). Their peak shapes were also complete with a favorable separation effect, and no interference peaks appeared.

### Linearity and range of calibration curves

**Fig. 3:**
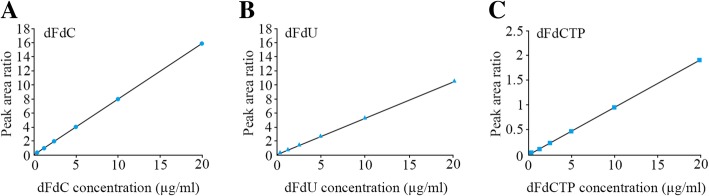
Linearity plots of dFdC (**a**), dFdU (**b**) and dFdCTP (**c**)

The average of peak areas of dFdC, dFdU and dFdCTP was analyzed using least square linear regression as a function of analyte concentration. We found all drugs had a good linear relationship in the concentration range of 0.5–20.0 μg/ml, with a correlation coefficient (r^2^) of ≥0.9998 (Fig. 3). Several parameters of regression curves are displayed in Table 2, including their slopes and intercepts for all pure drugs.

**Table 2:** Linear regression parameters of calibration equations for the standards

Drugs	Analytical wavelength	Linearity (μg/ml)	Correlation coefficient (r^2^)	Slope	Intercept
dFdC	268 nm	0.5–20.0	0.9999	0.7811	0.1209
dFdU	268 nm	0.5–20.0	0.9999	0.5112	0.0650
dFdCTP	268 nm	0.5–20.0	0.9998	0.0955	0.0014

### Precision and accuracy of the adopted method

The precision of the given scheme was confirmed by analyzing QC samples at 0.5, 5.0and 10.0 μg/ml for all target compounds. The results are expressed as % RSD (Table 3). We found the range of intra-day variations was 1.42–5.17%. The inter-day variations were smaller than 9.02% under different drug concentrations. In light of this, variations to this extent in the RP-HPLC method results were considered acceptable. Average accuracy observations revealed that this method was precise with a percentage recovery of 88.04–98.28%. This met the detection criteria.

**Table 3:** Intra-day and inter-day precision and average accuracy

Standard component	Concentration (μg/ml)	Average accuracy (%Recovery)	Intra-day precision (%RSD)	Inter-day precision (%RSD)
dFdC	0.5	94.71 ± 0.03	4.89	6.54
	5.0	93.49 ± 0.05	4.51	5.72
	10.0	98.28 ± 0.02	3.09	4.65
dFdU	0.5	88.04 ± 0.07	4.00	9.02
	5.0	90.91 ± 0.04	3.79	7.89
	10.0	96.08 ± 0.11	3.65	4.49
dFdCTP	0.5	90.86 ± 0.03	5.17	8.15
	5.0	93.85 ± 0.06	3.96	9.02
	10.0	95.60 ± 0.08	1.42	4.28

### LOD and LOQ of three standards

According to the standard deviation of the Y-intercepts of the different calibration lines and two specified calculation formulas, LOD was found to be 0.2063, 0.3151and 1.6870 μg/ml for dFdC**,** dFdU and dFdCTP, respectively, while LOQ was 0.6250, 0.9550 and 5.1120 μg/ml.

### Concentration changes of dFdC, dFdU and dFdCTP

**Fig. 4:**
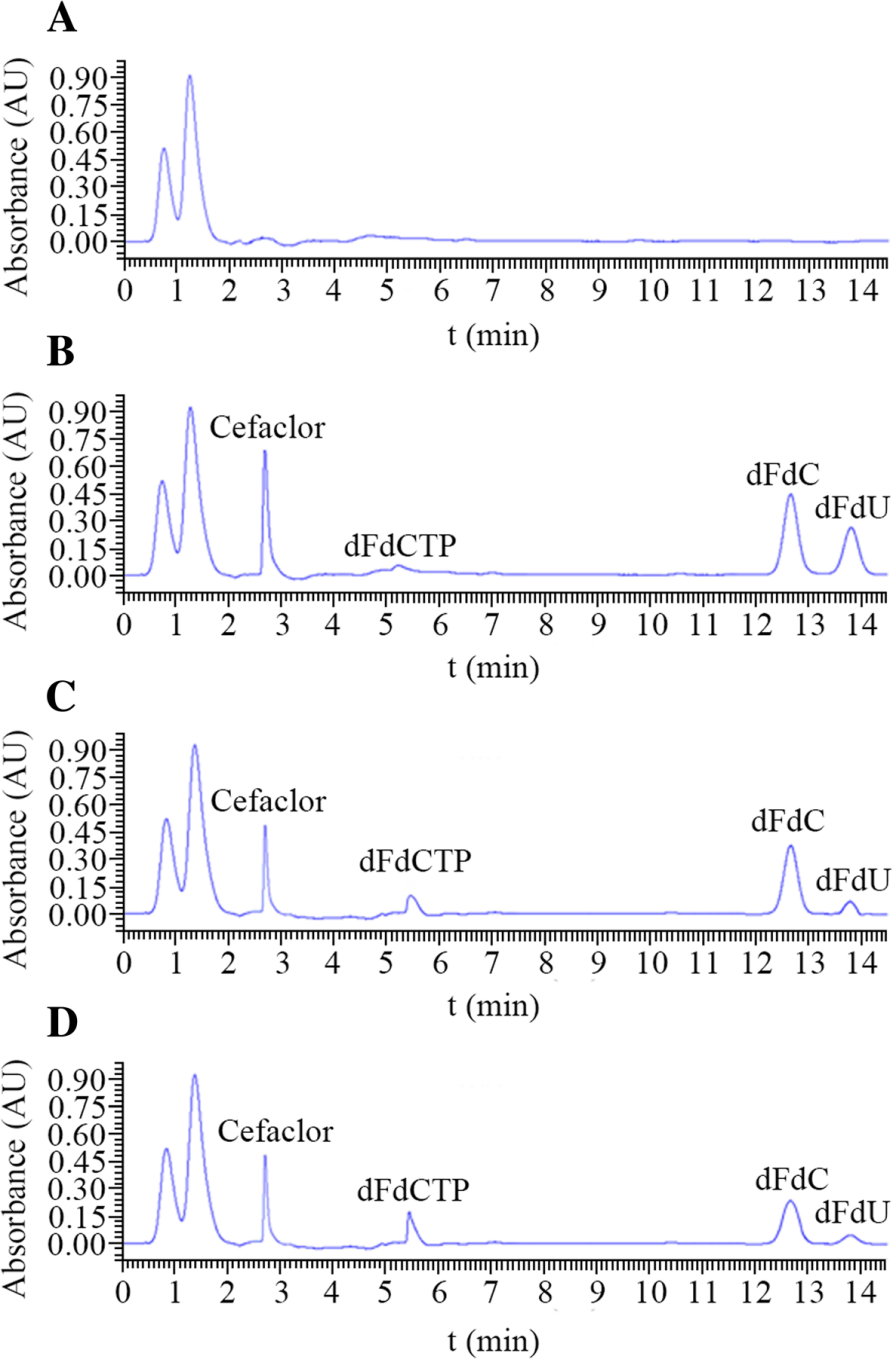
Representative chromatograms (268 nm) of PANC-1 cell lysates after metabolism under differentconditions of no drugs (**a**) or loading dFdC at IC_50_ along with a heat treatment of 37 °C (**b**), 41 °C (**c**) or 43 °C (**d**)

**Fig. 5:**
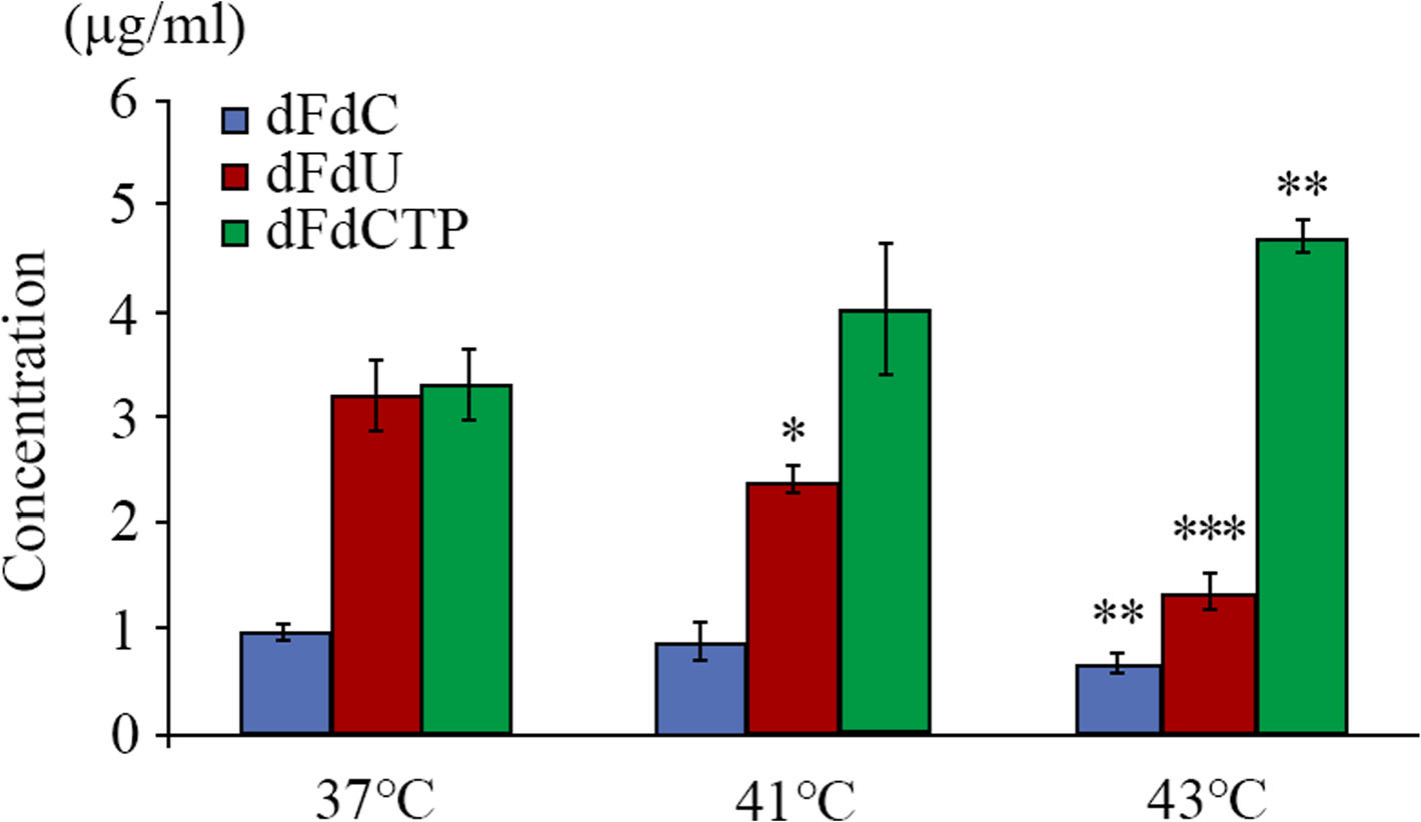
Effects of hyperthermia on concentration changes in dFdC, dFdU and dFdCTP. Error bars indicate standard errors. *n* = 5, **p < 0.05, **p < 0.01, ***p < 0.001

After the PANC-1 cells in the logarithmic growth phase were treated with dFdC combined with hyperthermia, the concentrations of dFdC and its metabolites had changed to some extent (Table 4, Figs. 4 and 5). In the groups of dFdC combined with 41 °C or 43 °C hyperthermia, dFdC was observed to decrease 9.28 and 30.93%,respectively;dFdU showed a significant downward trend (i.e., 24.76 and 57.80%); and dFdCTP increased 21.82 and 42.42%.

**Table 4:** Effects of hyperthermia on changes in the concentration of dFdC and its metabolites

	dFdC (μg/ml)	dFdU (μg/ml)	dFdCTP (μg/ml)
37 **°**C	1.02	2.86	3.24
	0.91	3.65	3.25
	1.03	3.41	3.33
	0.90	2.91	2.89
	1.02	3.15	3.81
Average value	0.97	3.19	3.30
Standard deviation	0.063	0.33	0.34
41 **°**C	1.12	2.29	4.17
	0.97	2.57	3.92
	0.70	2.33	4.98
	0.94	2.52	3.37
	0.66	2.31	3.68
Average value	0.88	2.40	4.02
Standard deviation	0.19	0.13	0.61
43 **°**C	0.82	1.43	4.79
	0.68	1.46	4.67
	0.64	1.14	4.76
	0.69	1.15	4.81
	0.55	1.52	4.46
Average value	0.67	1.34	4.70
Standard deviation	0.099	0.18	0.14

Based on the above results, we believe that the two heat treatments could alter the metabolic process of dFdC in PANC-1 cells, with a greater effect from43 °C hyperthermia. With regard to the decrease in dFdC content, one main explanation is that hyperthermia could promote the process of converting dFdC into its metabolites (e.g., dFdCTP). In addition, we cautiously predict the decrease in dFdU content is most likely due to a reduction of the activity of cytidine deaminase (CDA) caused by hyperthermia, thereby inhibiting the direct and (or) indirect transformation from dFdC to dFdU. It is known that dFdU is an inactive metabolite of irreversible deamination of dFdCTP [10], which occurs rapidly under the action of CDA, which plays an indispensable role in the detoxification of dFdC [32, 33].

Surprisingly, dFdC combined with 43 °C hyperthermia was more conducive to the production of dFdCTP, which is an active ingredient produced by dFdC in cells after the action of deoxycytidine kinase (dCKs) and nucleoside kinase [34]. Relevant studies have shown that dCKs are the rate-limiting enzyme of dFdC, and its activity is closely related to chemosensitivity [35]. Therefore, we speculate that hyperthermia increases the activity of dCKs and this may be viewed as a possible explanation for the rising dFdCTP level. On the other hand, we consider that a decrease in dFdCTP consumption owing to a reduction in CDA activity and the activation of dCKs by the intermediate of dFdCDP may also partly contribute to the increase in this active substance. Certainly, the definite response mechanism of hyperthermia leading to content variations in dFdC and its metabolites still needs further exploration.

## Conclusions

In this study, we used an optimized RP-HPLC assay to show that variances in the concentration of dFdC (gemcitabine) and its metabolites occurred in PANC-1 cells when pretreated with 41 °C and 43 °C hyperthermia. We also confirmed that 43 °C hyperthermiaha sa more significant effect on the drug metabolism. However, the molecular mechanisms leading to the observed effects are still unclear. This initial study of the effects of two heat treatments on dFdC metabolism should provide a theoretical basis for further research of its pharmacokinetic mechanisms and offer a reference for the best regimen of this combination therapy for pancreatic cancer clinical treatments.

## Abbreviations

dCMP: 2′-deoxycytidine 5′-monophosphate
dFdC: 2′,2′-difluoro-2′-deoxycytidine, Gemcitabine
dFdCTP: difluorodeoxycytidine triphosphate
dFdU: 2′,2′-difluorodeoxyuridine
RP-HPLC: reverse phase high-performance liquid chromatography

## References

[1] Siegel RL, Miller KD, Jemal A (2017). Cancer statistics, 2017. CA Cancer J Clin.

[2] Miller KD, Siegel RL, Lin CC, Mariotto AB, Kramer JL, Rowland JH (2016). Cancer treatment and survivorship statistics, 2016. CA Cancer J Clin.

[3] Rahib L, Smith BD, Aizenberg R, Rosenzweig AB, Fleshman JM, Matrisian LM (2014). Projecting Cancer incidence and deaths to 2030: the unexpected burden of thyroid, liver, and pancreas cancers in the United States. Cancer Res.

[4] Enewold LHL, Tucker T, McKenzie S (2015). Pancreatic cancer in the USA: persistence of undertreatment and poor outcome. Journal of gastrointestinal cancer.

[5] Eppinga W, Lagerwaard F, Verbakel W (2010). Volumetric modulated arc therapy for advanced pancreatic Cancer. Strahlenther Onkol.

[6] Ohguri T, Imada H, Yahara K (2008). Concurrent chemoradiotherapy with gemcitabine plus regional hyperthermia for locally advanced pancreatic carcinoma: initial experience [J]. Radiat Med.

[7] Konski A, Meyer JE, Joiner M, Hall MJ, Philip P, Shields A (2014). Multi-institutional phase I study of low-dose ultra-fractionated radiotherapy as a chemosensitizer for gemcitabine and erlotinib in patients with locally advanced or limited metastatic pancreatic cancer. Radiother Oncol.

[8] Burris HA, Moore MJ, Andersen J, Green MR, Rothenberg ML, Modiano MR (1997). Improvements in survival and clinical benefit with gemcitabine as first-line therapy for patients with advanced pancreas cancer: a randomized trial. J Clin Oncol.

[9] Valsecchi ME, Díaz-Cantón E, de la Vega M, Littman SJ (2014). Recent treatment advances and novel therapies in pancreas cancer: a review. Journal of gastrointestinal cancer..

[10] Wickremsinhe E, Bao J, Smith R, Burton R, Dow S, Perkins E (2013). Preclinical absorption, distribution, metabolism, and excretion of an oral amide prodrug of gemcitabine designed to deliver prolonged systemic exposure. Pharmaceutics.

[11] Goldstein D, El-Maraghi RH, Hammel P, Heinemann V, Kunzmann V, Sastre J (2015). nab-Paclitaxel Plus Gemcitabine for Metastatic Pancreatic Cancer: Long-Term Survival From a Phase III Trial. JNCI: Journal of the National Cancer Institute.

[12] Von Hoff DD, Ervin T, Arena FP, Chiorean EG, Infante J, Moore M (2013). Increased survival in pancreatic cancer with nab-paclitaxel plus gemcitabine [J]. N Engl J Med.

[13] Zhang DSWD, Wang ZQ, Wang FH, Luo HY, Qiu MZ, Wang F, Li YH, Xu RH (2013). Phase I/II study of albumin-bound nab-paclitaxel plus gemcitabine administered to Chinese patients with advanced pancreatic cancer. Cancer Chemother Pharmacol.

[14] Suriyanto, EYK N, Kumar SD (2017). Physical mechanism and modeling of heat generation and transfer in magnetic fluid hyperthermia through Néelian and Brownian relaxation: a review. Biomedical engineering online.

[15] van der Zee J (2002). Heating the patient: a promising approach?. Annals of Oncology.

[16] Song CW, Park HJ, Lee CK, Griffin R (2005). Implications of increased tumor blood flow and oxygenation caused by mild temperature hyperthermia in tumor treatment. Int J Hyperth.

[17] Issels RD (2008). Hyperthermia adds to chemotherapy. Eur J Cancer.

[18] BAKSHANDEH-BATH A, STOLTZ AS, HOMANN N, WAGNER T, STÖLTING S, PETERS SO (2009). Preclinical and clinical aspects of carboplatin and gemcitabine combined with whole-body hyperthermia for pancreatic adenocarcinoma. Anticancer Res.

[19] Tentes A-A, Stamou K, Pallas N, Karamveri C, Kyziridis D, Hristakis C (2016). The effect of hyperthermic intraoperative intraperitoneal chemotherapy (HIPEC) as an adjuvant in patients with resectable pancreatic cancer. Int J Hyperth.

[20] Adachi S, Kokura S, Okayama T, Ishikawa T, Takagi T, Handa O (2009). Effect of hyperthermia combined with gemcitabine on apoptotic cell death in cultured human pancreatic cancer cell lines. Int J Hyperth.

[21] Guo Y, Ziesch A, Hocke S, Kampmann E, Ochs S, De Toni EN (2015). Overexpression of heat shock protein 27 (HSP27) increases gemcitabine sensitivity in pancreatic cancer cells through S-phase arrest and apoptosis. J Cell Mol Med.

[22] Adema AD, Smid K, Losekoot N, Honeywell RJ, Verheul HM, Myhren F (2012). Metabolism and accumulation of the lipophilic deoxynucleoside analogs elacytarabine and CP-4126[J]. Investig New Drugs.

[23] Giovannetti E, Del Tacca M, Mey V, Funel N, Nannizzi S, Ricci S (2006). Transcription analysis of human Equilibrative nucleoside Transporter-1 predicts survival in pancreas Cancer patients treated with gemcitabine. Cancer Res.

[24] Farrell JJ, Elsaleh H, Garcia M, Lai R, Ammar A, Regine WF (2009). Human Equilibrative nucleoside transporter 1 levels predict response to gemcitabine in patients with pancreatic Cancer. Gastroenterology..

[25] Ishikawa T, Kokura S, Sakamoto N, Ando T, Imamoto E, Hattori T (2012). Phase II trial of combined regional hyperthermia and gemcitabine for locally advanced or metastatic pancreatic cancer. Int J Hyperth.

[26] Affram K, Udofot O, Cat A, Agyare E (2015). In vitro and in vivo antitumor activity of gemcitabine loaded thermosensitive liposomal nanoparticles and mild hyperthermia in pancreatic cancer. Int J Adv Res.

[27] Veltkamp SA, Pluim D, van Eijndhoven MAJ, Bolijn MJ, Ong FHG, Govindarajan R (2008). New insights into the pharmacology and cytotoxicity of gemcitabine and 2′,2′-difluorodeoxyuridine. Mol Cancer Ther.

[28] Sugarbaker PH, Stuart OA, Bijelic L. Intraperitoneal Gemcitabine Chemotherapy Treatment for Patients with Resected Pancreatic Cancer: Rationale and Report of Early Data 2011. 7 p. 10.1155/2011/161862 PMC3263652 22312494

[29] Kumar KK, Nagoji KEV, Nadh RV (2012). A validated RP-HPLC method for the estimation of Lapatinib in tablet dosage form using gemcitabine hydrochloride as an internal standard. Indian J Pharm Sci.

[30] Shyam SB, Christian C, Silvia F, Erika Z, Mauro F, Ganesh P (2013). Validated RP-HPLC method for the simultaneous analysis of gemcitabine and LY-364947 in liposomal formulations. Curr Drug Targets.

[31] Lanz C, Früh M, Thormann W, Cerny T, Lauterburg BH (2007). Rapid determination of gemcitabine in plasma and serum using reversed-phase HPLC. J Sep Sci.

[32] Yonemori K, Ueno H, Okusaka T, Yamamoto N, Ikeda M, Saijo N (2005). Severe drug toxicity associated with a single-nucleotide polymorphism of the <em>cytidine deaminase</em> gene in a Japanese Cancer patient treated with gemcitabine plus cisplatin. Clin Cancer Res.

[33] Ogawa M, Hori H, Ohta T, Onozato K, Miyahara M, Komada Y (2005). Sensitivity to gemcitabine and its metabolizing enzymes in neuroblastoma. Clin Cancer Res.

[34] Veltkamp SA, Beijnen JH, Schellens JHM (2008). Prolonged versus standard gemcitabine infusion: translation of molecular pharmacology to new treatment strategy. Oncologist.

[35] Maréchal R, Mackey JR, Lai R, Demetter P, Peeters M, Polus M (2010). Deoxycitidine kinase is associated with prolonged survival after adjuvant gemcitabine for resected pancreatic adenocarcinoma. Cancer.

